# Mesenchymal stromal cells plus basiliximab improve the response of steroid-refractory acute graft-versus-host disease as a second-line therapy: a multicentre, randomized, controlled trial

**DOI:** 10.1186/s12916-024-03275-5

**Published:** 2024-02-27

**Authors:** Haixia Fu, Xueyan Sun, Ren Lin, Yu Wang, Li Xuan, Han Yao, Yuanyuan Zhang, Xiaodong Mo, Meng lv, Fengmei Zheng, Jun Kong, Fengrong Wang, Chenhua Yan, Tingting Han, Huan Chen, Yao Chen, Feifei Tang, Yuqian Sun, Yuhong Chen, Lanping Xu, Kaiyan Liu, Xi Zhang, Qifa Liu, Xiaojun Huang, Xiaohui Zhang

**Affiliations:** 1 Peking University People’s Hospital, Peking University Institute of Haematology, No. 11 Xizhimen South Street, Beijing, 100044 China; 2 Collaborative Innovation Center of Haematology, Peking University, Beijing, China; 3 Beijing Key Laboratory of Haematopoietic Stem Cell Transplantation, Beijing, China; 4 National Clinical Research Center for Haematologic Disease, Beijing, China; 5 Medical Center of Haematology, State Key Laboratory of Trauma, Burn and Combined Injury, Xinqiao Hospital, Army Medical University, Chongqing, 400037 China; 6 Department of Haematology, Nanfang Hospital, Southern Medical University, Guangzhou, 510515 China

**Keywords:** Mesenchymal stromal cells, Haemopoietic stem cell transplantation, Haploidentical, Acute graft-versus-host disease, Steroid-refractory, Second-line therapy

## Abstract

**Background:**

For patients with steroid-refractory acute graft-versus-host disease (SR-aGVHD), effective second-line regimens are urgently needed. Mesenchymal stromal cells (MSCs) have been used as salvage regimens for SR-aGVHD in the past. However, clinical trials and an overall understanding of the molecular mechanisms of MSCs combined with basiliximab for SR-aGVHD are limited, especially in haploidentical haemopoietic stem cell transplantation (HID HSCT).

**Methods:**

The primary endpoint of this multicentre, randomized, controlled trial was the 4-week complete response (CR) rate of SR-aGVHD. A total of 130 patients with SR-aGVHD were assigned in a 1:1 randomization schedule to the MSC group (receiving basiliximab plus MSCs) or control group (receiving basiliximab alone) (NCT04738981).

**Results:**

Most enrolled patients (96.2%) received HID HSCT. The 4-week CR rate of SR-aGVHD in the MSC group was obviously better than that in the control group (83.1% vs. 55.4%, *P* = 0.001). However, for the overall response rates at week 4, the two groups were comparable. More patients in the control group used ≥ 6 doses of basiliximab (4.6% vs. 20%, *P* = 0.008). We collected blood samples from 19 consecutive patients and evaluated MSC-derived immunosuppressive cytokines, including HO1, GAL1, GAL9, TNFIA6, PGE2, PDL1, TGF-β and HGF. Compared to the levels before MSC infusion, the HO1 (*P* = 0.0072) and TGF-β (*P* = 0.0243) levels increased significantly 1 day after MSC infusion. At 7 days after MSC infusion, the levels of HO1, GAL1, TNFIA6 and TGF-β tended to increase; however, the differences were not statistically significant. Although the 52-week cumulative incidence of cGVHD in the MSC group was comparable to that in the control group, fewer patients in the MSC group developed cGVHD involving ≥3 organs (14.3% vs. 43.6%, *P* = 0.006). MSCs were well tolerated, no infusion-related adverse events (AEs) occurred and other AEs were also comparable between the two groups. However, patients with malignant haematological diseases in the MSC group had a higher 52-week disease-free survival rate than those in the control group (84.8% vs. 65.9%, *P* = 0.031).

**Conclusions:**

For SR-aGVHD after allo-HSCT, especially HID HSCT, the combination of MSCs and basiliximab as the second-line therapy led to significantly better 4-week CR rates than basiliximab alone. The addition of MSCs not only did not increase toxicity but also provided a survival benefit.

**Supplementary Information:**

The online version contains supplementary material available at 10.1186/s12916-024-03275-5.

## Background

Severe acute graft-versus-host disease (aGVHD) strongly predicts the poor prognosis of allogeneic haemopoietic stem cell transplantation (allo-HSCT) [1, 2] and causes death in 16–19% of adult allo-HSCT patients [3]. Steroids are a recognized first-line treatment for aGVHD, yet 50% of aGVHD cases are steroid-refractory [4–6], known as steroid-refractory aGVHD (SR-aGVHD). After the failure of steroids, second-line options for SR-aGVHD mainly consist of ruxolitinib [7], interleukin-2 receptor (IL-2R) blockade [8, 9], extracorporeal photopheresis (ECP), methotrexate (MTX) and mycophenolate mofetil (MMF) [10]. However, due to the lack of well-designed trials to compare the efficacy between second-line treatment approaches, optimal second-line treatments have not been established. Guidelines for aGVHD recommend that centres should follow their institutional guidelines to choose second-line treatments for SR-aGVHD. Basiliximab is an important IL-2 receptor (IL-2R) blockade agent that can inhibit the rapid proliferation of activated T lymphocytes by binding to the α-chain of IL-2R [10]. The efficacy of basiliximab in SR-aGVHD has been demonstrated in previous single-arm prospective studies with a limited sample size [9]. However, the inhibition of IL-2R may not only inhibit activated T lymphocytes but also reduce Treg proliferation and may be one reason for the unsatisfactory efficacy of inhibitors of IL-2R used alone in the treatment of aGVHD [11]. In addition, in SR aGVHD, steroids combined with calcineurin inhibitors (CNIs) may impair both effector T cells and regulatory T cells, and damaged regulatory T cells may lead to the failure of immune tolerance. Long-term steroid exposure may also perpetuate inflammation, and in GI SR-aGVHD, steroids may inhibit the repair of host tissue injury mediated by T cells [12]. Therefore, new therapeutic agents improving the curative effect of basiliximab by enhancing Treg proliferation and promoting tissue repair are urgently needed.

Mesenchymal stromal cells (MSCs), which can stimulate an immunosuppressive and immunoregulatory environment, limit tissue damage, support haematopoiesis and promote tissue repair, are potential therapies for SR-aGVHD [13]. Previous studies found that MSC-treated SR-aGVHD patients exhibited higher Treg numbers and frequencies, which was one of the mechanisms for the treatment of SR-aGVHD [13, 14]. Aside from immunomodulation, MSCs have also been shown to play a strong role in supporting haematopoiesis, limiting tissue damage and stimulating tissue repair, which are crucial in the treatment of SR-aGVHD [13, 15, 16]. This may be the rationale for using MSCs in combination with basiliximab as second-line therapy for SR-aGVHD. Furthermore, increasing evidence has shown that the therapeutic benefit of MSCs is mainly attributed to the regulation of innate and adaptive immunity via the secretion of chemokines, cytokines, extracellular vesicles and growth factors. Various soluble factors, including haem oxygenase-1 (HO-1), TNFα stimulated gene 6 (TSG-6), prostaglandin E2 (PGE2) and transforming growth factor (TGF-β), have been demonstrated to be involved in MSC modulation of immune systems by exerting immunomodulatory effects on a wide variety of immune cells. For example, PGE2 inhibits the maturation, activation and antigen presentation of dendritic cells and the proliferation of T cells [17]. TGF-β also suppresses the activation and proliferation of T cells [18]. Thus, MSC therapies have been used for many inflammatory and autoimmune diseases, such as asthma, colitis and GVHD.

With the combination of MSCs and basiliximab, the combined effects on effector T cells, regulatory T cells, other lymphocytes and cytokines may lead to a better response in the treatment of SR-aGVHD, which may be the rationale for using MSCs in combination with basiliximab as second-line therapy for SR-aGVHD. However, the relevant evidence is limited. Few reports have shown changes in the immunomodulatory factors of MSCs in this setting, which may indicate the molecular mechanisms of these cells.

In recent years, device studies have evaluated the clinical use of MSCs for SR-aGVHD, and most of these trials have shown that combining MSCs with other second-, third- or multiline drugs as treatment for SR-aGVHD is safe and effective, with response rates ranging from 33 to 100% [19–23]. However, the evidence for the combination of MSCs and basiliximab used to treat SR-aGVHD is limited. The studies mentioned above have mostly focused on the clinical effect of MSCs on SR-aGVHD, and few reports have shown changes in the immunomodulatory factors of MSCs in this setting, which may indicate the molecular mechanisms of these cells.

To determine whether the regimen of MSCs combined with basiliximab could result in a superior efficacy and safety profile compared to basiliximab alone as the second-line therapy for SR-aGVHD, this multicentre, randomized controlled trial was conducted in adult allo-HSCT recipients (NCT04738981). We also analysed the changes in MSC-derived cytokines after MSC treatment to obtain mechanistic insights into the clinical applications.

## Methods

### Study design and patients

This multicentre, randomized, controlled, prospective trial was conducted in 3 hospitals (Peking University People’s Hospital, Nanfang Hospital and Xinqiao Hospital) according to the Declaration of Helsinki from February 1, 2021, to May 2, 2022. The protocol of this trial was approved by the ethics committees of the 3 hospitals and was registered as NCT04738981 at ClinicalTrials.gov.

Eligible patients were aged 18–70 years and diagnosed with SR-aGVHD after allo-HSCT. The presence of SR-aGVHD was defined as treatment with ≥ 1 mg/kg/day of methylprednisolone or equivalent, aGVHD continuing to progress after 3 days, no response after 7 days or exacerbation during steroid tapering [5, 24–26]. Other eligibility criteria included patients who achieved neutrophil engraftment and exhibited creatinine levels below 2 times the normal upper limit at enrolment. The enrolled patients had to be willing and able to sign written informed consent. Patients were excluded if they could not tolerate the treatment; had advanced or relapsed primary disease; presented active bacterial, fungal or viral infections; exhibited dysfunction of multiple organs; or were deemed unsuitable for the study according to the investigator’s evaluation. Female patients who were pregnant or lactating or who planned pregnancy during the study period were also ineligible.

### Randomization and masking

Using a central, interactive web-based system, enrolled patients were assigned in a 1:1 randomization schedule to the MSC group (receiving basiliximab plus MSCs) or control group (receiving basiliximab alone). Randomization was stratified according to the patients’ sex. In this open-label study, clinicians and patients were not blinded to the group assignments, but the experts who participated in the data collection and analysis were.

### Transplant procedures

During allo-HSCT, HLA typing, donor selection, infection and GVHD prevention, and supportive care were performed according to our previous reports [27–29]. For patients with malignant haematologic diseases, the BU/CY-based conditioning protocol comprised the following: (1) in HID HSCT: cytarabine (4 g/m^2^ per day on days −10 to −9); busulfan (3.2 mg/kg per day on days −8 to −6); cyclophosphamide (1.8 g/m^2^ per day on days −5 to −4); methyl chloride hexamethylene urea nitrate (Me-CCNU, 250 mg/m^2^ per day on day −3); thymoglobulin (ATG, 2.5 mg/kg per day on days −5 to −2, Sang Stat, Lyon, France); (2) in unrelated donor (URD) HSCT: cytarabine (2 g/m^2^ per day on days −10 to −9); other regimens were the same as type (1); (3) in matched identical sibling donor (MSD) HSCT: hydroxycarbamide (80 mg/kg) orally on day −10; cytarabine (2 g/m^2^ per day on day −9); with ATG (1.5 mg/kg per day on days −5 to −3 for patients ≥40 years). In BU/FLU-based conditioning therapy, fludarabine (30 mg/m^2^ on day −5 to −2) replaced cyclophosphamide; in TBI-based conditioning therapy, total body irradiation (TBI, 700 cGy) replaced cytarabine and BU; and the other regimens were the same as the BU/CY-based regimens. In HID HSCT and URD HSCT for SAA, patients received a BU/CY-based regimen, which consisted of busulfan (3.2 mg/kg per day on days −7 to −6), cyclophosphamide (50 mg/kg per day on days −5 to −2) and antithymoglobulin (ATG, 2.5 mg/kg per day on days −5 to −2, Sang Stat, Lyon, France). In MSD HSCT, busulfan was not administered, and the other regimens were the same as those described above.

For the prevention of infections, all patients received prophylactic antibiotics, antifungals and antiviral drugs from the start of conditioning [30]. For GVHD prophylaxis, cyclosporine A (CsA), MMF and short-term MTX were administered [31, 32]. Patients who received transplants from maternal donors or relative donors (e.g. uncle, aunt, nephew, niece and cousins) also received low-dose posttransplant cyclophosphamide (PTCy) (14.5 mg/kg on days 3 and 4) to strengthen the prevention of GVHD [31, 32]. The details of prophylaxis for infection and GVHD are shown in Additional files 1 and 2.

### MSC preparation

In this trial, we used MSCs isolated from human umbilical cord blood (UC-MSCs), which were prepared by the Beijing Engineering Lab for Cell Therapy (Beijing, China). The isolation, culture, identification and infusion of UC-MSCs were performed according to previous studies. In brief, the umbilical cord segments were dissected into small pieces (1–3 mm^3^) and cultured in a serum-free culture system, in which UltraGRO™-Advanced (HPCFDCRL50, Helios Bioscience, America) was used for platelet lysis. The culture medium was changed every week until the cells were expanded and reached subconfluence (80%). After pouring out the culture medium, the culture flask was washed with 0.9% normal saline twice, and then 1.5 mL of trypsin (0.125%) was added to the flask for cell digestion. When the cells became round and suspended, digestion was stopped by adding 3 mL of medium. After centrifuging and cleaning, the cell suspension was replated in the flask with the same culture conditions for passage. Cells were passaged until P5. Then, the cells were conserved for clinical use if they met the following criteria: (1) MSCs were within the 5th generation or the cell count was < 5 × 10^7^ (100 mL); (2) MSCs were spindle-shaped or fibroblast-like in vitro culture; (3) molecular markers were CD73^+^/CD90^+^/CD105^+^ ≥ 95% and CD34^+^/HLA-DR^+^/CD45^+^⩽2%; (4) no viral, fungal, bacterial, or mycoplasma contamination, endotoxin ≤ 0.5 EU/mL; and (5) live cell ratio ≥ 90% before infusion and completion of infusion within 18 h from discharge.

### Treatment protocol

**Fig. 1:**
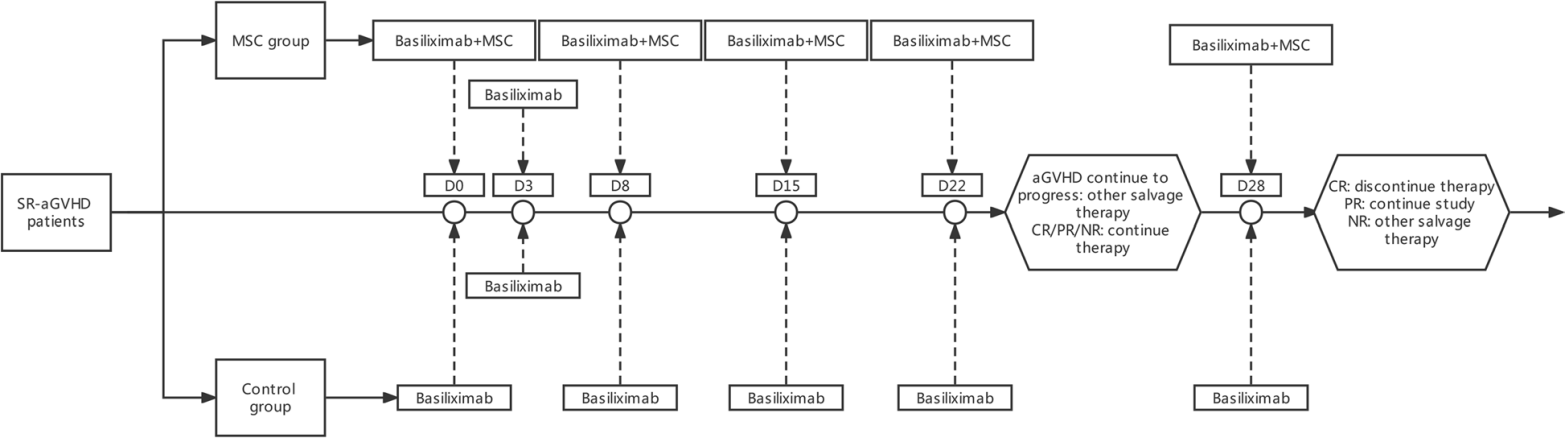
Treatment protocol for eligible patients. Patients randomly assigned to the MSC group received MSCs plus basiliximab, and the control group received basiliximab alone. MSCs were administered once a week for 4 weeks as a cycle. Further administrations of MSCs were given if the patients achieved PR at week 4. Basiliximab was administered twice in the first week and once a week until aGVHD was reduced to grade < II. MSCs, mesenchymal stromal cells; CR, complete response; PR, partial response; NR, no response

Patients randomly assigned to the MSC group received both MSCs and anti-CD25 mAb (basiliximab), or the control group received basiliximab alone. The details were as follows: MSCs (1.0 × 10^6^/kg) were infused once a week for 4 weeks as a cycle. If patients achieved a partial response (PR) after the first 4 weeks of treatment, the MSC infusions were repeated in another 4 weeks. Basiliximab (20 mg) was administered on days 1 and 3 and once a week thereafter until aGVHD was reduced to < grade II. However, if SR-aGVHD continued to progress within 3 weeks of treatment or lacked a response after 4 weeks of treatment, the patients could be switched to other salvage treatments for SR-aGVHD (Fig. 1). In both groups, the dose of steroids was tapered by 30% every 5 days and withdrawn within 4 weeks after two doses of basiliximab [33].

After randomization, study visits were scheduled at baseline, week 4, week 8, week 12, week 24 and week 52. At every visit, we collected data on the SR-aGVHD response, survival, chronic GVHD (cGVHD) [34] and safety outcomes, including relapse, infection, complete blood counts, renal function and liver function.

### Endpoints and assessments

SR-aGVHD responses were assessed based on the consensus criteria [5, 35]. For complete response (CR), the definition was the resolution of aGVHD in all involved organs. If the SR-aGVHD improved in either organ for at least one aGVHD stage and without any worsening in any other organ, patients were defined as PR. The overall response (OR) included both PR and CR. If patients could not achieve the criteria of PR, they were defined as having no response (NR).

The primary endpoint was the 4-week CR rate of SR-aGVHD. The major secondary endpoints were overall survival (OS) at the end of weeks 4/8/12/24/52. OS was defined as the time from randomization to death for any reason, the end of the study for patients who were alive or the last follow-up day for missing patients. Other secondary endpoints included the rate of PR at week 4 and the infusion toxicity of MSCs. The infusion toxicity of MSCs involved an acute toxicity response and a chronic toxicity response. The acute toxicity response meant toxicity leading to injury of the heart, kidney or liver within 4 h after MSC infusion, and the chronic toxicity response entailed secondary tumours and relapse of underlying haematologic malignancies.

Safety assessments were performed by physical examination and laboratory assessments to monitor the frequency and severity of adverse events (AEs), which involved acute and chronic infusion toxicity of MSCs; injury to the heart, kidney and liver; haematologic toxicity; CMV and EBV infection; etc. AEs were graded according to the Common Terminology Criteria for Adverse Events (version 5.0).

#### ELISA

Blood samples from patients in the MSC group were collected before and at 1 and 7 days after MSC infusion. Enzyme-linked immunosorbent assay (ELISA) kits (Elabscience Biotechnology Co., Ltd., Wuhan, China) were used to determine the levels of MSC-derived suppressive cytokines, including haem oxygenase-1 (HO-1), galectin-1 (Gal-1), galectin-9 (Gal-9), TNFα stimulated gene 6 (TSG-6), prostaglandin E2 (PGE2), transforming growth factor (TGF)-β1, programmed death ligand 1 (PDL1) and hepatocyte growth factor (HGF).

### Statistical analysis

Based on preliminary studies [5, 35], we hypothesized that the proportion of patients with a 4-week CR would increase from 60% for basiliximab monotherapy to 80% for MSCs plus basiliximab. Pearson’s *χ*^2^ test at the two-sided 0.05 level was used to determine the difference between treatment groups. To achieve 80% power and allow for 10% dropout, each treatment group needed 65 participants.

All participants undergoing randomization were included in the intention-to-treat (ITT) population, and baseline characteristics and primary and secondary endpoints were analysed in the ITT population. The modified ITT (mITT) population consisted of patients who survived less than 100 days after allo-HSCT, which were used to assess the incidence and severity of cGVHD. The safety analysis set consisted of patients receiving ≥ 1 dose of treatment in either group.

Pearson *χ*^2^ tests and Mann–Whitney *U* tests were used for categorical variables and continuous variables in the comparative analysis, respectively. Kaplan–Meier analysis was used to estimate the OS and DFS at the end of week 4/8/12/24/52. The hazard ratios (HRs) and 95% confidence intervals (CIs) were also calculated. Relapse and NRM were competing risks for disease-free survival (DFS). All statistical analyses were performed using SPSS 25.0 software or R software version 4.1.2. *P* < 0.05 for a two-sided test was considered statistically significant.

## Results

### Patient characteristics

**Fig. 2:**
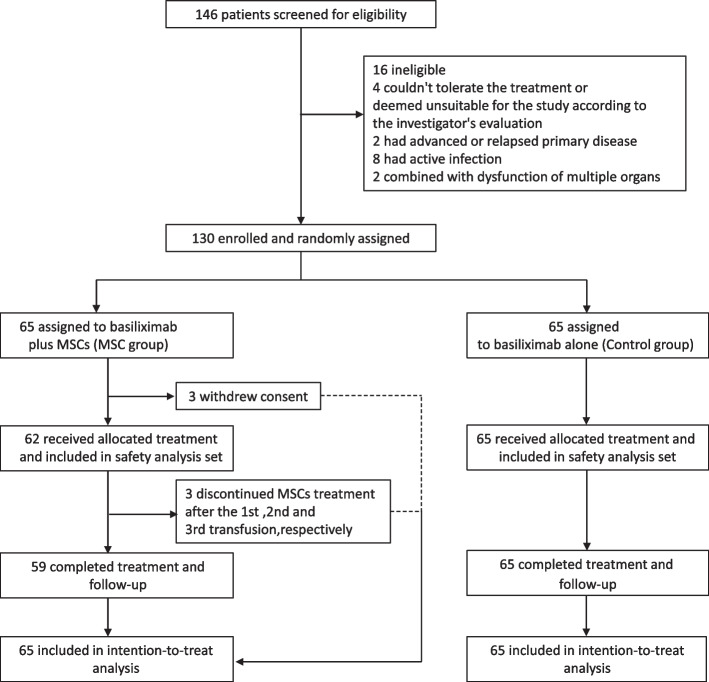
Flowchart of patients

A total of 146 adult patients with SR-aGVHD were screened for enrolment between Jan 1, 2021, and May 2, 2022. Among them, eight met the exclusion criteria and eight withdrew informed consent, and the remaining 130 participants were included in the ITT population and randomized to receive one of the two regimens: MSCs combined with basiliximab (MSC group, *n* = 65) or basiliximab monotherapy (control group, *n* = 65). After randomization, 3 patients withdrew consent due to their own decision, and they did not receive MSC infusions. Therefore, the safety analysis set excluded 3 patients. Figure 2 shows the flow diagram of this trial.

The ITT population comprised 68 male and 62 female patients with a median age of 32 years (range, 18–68). Most of them had acute myeloid leukaemia (AML, *n* = 49, 37.7%) and acute lymphoblastic leukaemia (ALL, *n* = 32, 24.6%). Most patients (*n* = 125, 96.2%) received HID HSCT for malignant or nonmalignant diseases, and the majority of the stem cell sources were PBSCs (*n* = 113, 86.9%). As shown in Table 1, there were no significant differences in baseline age, sex, underlying diseases or transplantation-related characteristics between the two groups.

**Table 1:** Baseline characteristics of patients with SR-aGVHD in the two groups

Characteristic	Total	MSC group (*n* = 65)	Control group (*n* = 65)	*P*
Age, median (range), years	32 (18–68)	33 (18–68)	31 (18–62)	0.633
Gender, *n* (%)				0.292
Male	68 (52.3%)	37 (56.9%)	31 (47.7%)	
Female	62 (47.7%)	28 (43.1%)	34 (52.3%)	
Disease, *n* (%)				0.275
AML	49 (37.7%)	26 (40.0%)	23 (35.4%)	
ALL	32 (24.6%)	15 (23.1%)	17 (26.15%)	
MDS	28 (21.5%)	11 (16.9%)	17 (26.15%)	
SAA	13 (10.0%)	6 (9.2%)	7 (10.8%)	
others	8 (6.2%)	7 (10.8%)	1 (1.5%)	
Donor type, *n* (%)				0.366
Haploidentical related donor		63 (96.92%)	62 (95.38%)	
Identical sibling donor		1 (1.54%)	3 (4.62%)	
Unrelated donor		1 (1.54%)	0 (0.00%)	
ABO blood type, *n* (%)				0.845
Matched		40 (61.5%)	39 (60.0%)	
Mismatched		25 (38.5%)	26 (40.0%)	
Conditioning regimen, *n* (%)				0.888
BU/CY-based	104 (80%)	51 (78.5%)	53 (81.5%)	
BU/FLU-based	20 (15.4%)	11 (16.9%)	9 (13.9%)	
TBI-based	6 (4.6%)	3 (4.6%)	3 (4.6%)	
GVHD prophylaxis				0.648
CsA, MMF, MTX+ low-dose PTCy	6 (4.6%)	2 (3.1%)	3 (4.6%)	
CsA, MMF, MTX	124 (95.4%)	63 (96.9%)	62 (95.4%)	
Transplanted total nucleated cell dose, ×10^8^/kg, median (range)		9.67 (6.07–16.60)	10.17 (5.52–18.93)	0.193
Transplanted CD34^+^ cell dose (×10^6^/kg, median, range)		3.24 (1.10–8.66)	2.71 (0.71–7.50)	0.065
Stem cell source				0.193
PBSCs	113 (86.9%)	59 (90.8%)	54 (83.1%)	
PBSCs+BM	17 (13.1%)	6 (9.2%)	11 (16.9%)	
Patients achieved WBC engraftment, *n* (%)				
Patients achieved PLT engraftment before day 90, *n* (%)		59 (90.77%)	55 (84.62%)	0.297
WBC engraftment time (post-HSCT days)		12 (8–19)	12 (11–20)	0.137
PLT engraftment time (post-HSCT days)^a^		11 (8–75)	13 (9–80)	0.221

*Abbreviations: AML* acute myelocytic leukaemia, *ALL* acute lymphoblastic leukaemia, *BM* bone marrow, *BU* busulfan, *CsA* cyclosporine A, *CY* cyclophosphamide, *FLU* fludarabine, *MDS* myelodysplastic syndrome, *MMF* mycophenolate mofetil, *MTX* methotrexate, *PLT* platelet, *PBSCs* peripheral blood stem cells, *PTCy* post-transplant cyclophosphamide, *SAA* severe aplastic anaemia, *TBI* total-body irradiation, *WBC* white blood cell

^a^For patients achieving platelet engraftment

### Characteristics of aGVHD

All aGVHDs were diagnosed at a median of 22 (range, 7–111) days after allo-HSCT. The duration from the occurrence of aGVHD to enrolment was 4 (range, 3 to 27) days. Of the 130 enrolled patients, 63 had grade II aGVHD, 34 had grade III aGVHD and 33 had grade IV aGVHD. Regarding the organs involved, 106 (81.5%) patients developed skin aGVHD, 90 (69.2%) developed gastrointestinal (GI) aGVHD and 32 (24.6%) developed liver aGVHD. The number of affected organs was 1 in 56 patients, 2 in 48 patients and 3 in 26 patients. The baseline characteristics of aGVHD were notably similar between the two groups, as shown in Table 2.

**Table 2:** Characteristics of SR-aGVHD on the time of enrollment

Characteristics of aGVHD	Total (*n* = 130)	MSC group (*n* = 65)	Control group (*n* = 65)	*P*
Severity of aGVHD, *n* (%)				0.921
Grade II	63 (48.5%)	32 (49.2%)	31 (47.7%)	
Grade III	34 (26.1%)	16 (24.6%)	18 (27.7%)	
Grade IV	33 (25.4%)	17 (26.2%)	16 (24.6%)	
Site of aGVHD, *n* (%)				
Skin	106 (81.5%)	55 (84.6%)	53 (81.5%)	0.64
GI	90 (69.2%)	42 (70.8%)	48 (73.8%)	0.254
Liver	32 (24.6%)	15 (23.1%)	17 (26.2%)	0.684
Number of sites, *n* (%)				0.888
1	56 (43.1%)	28 (53.1%)	28 (53.1%)	
2	48 (36.9%)	25 (38.5%)	23 (35.4%)	
3	26 (20.0%)	12 (18.5%)	14 (21.5%)	

*Abbreviations: aGVHD* acute graft-versus-host disease, *GI* gastrointestinal

### MSCs improved the CR rate of SR-aGVHD at week 4

**Fig. 3:**
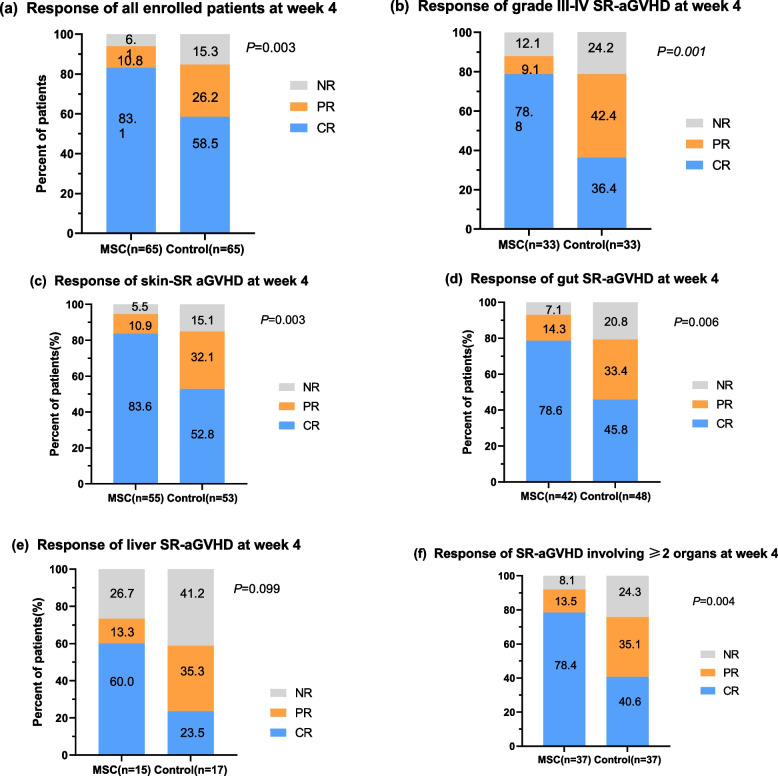
Response rates of SR-aGVHD after 4 weeks of treatment. Patients in the MSC group (using MSCs plus basiliximab) achieved superior responses compared to outcomes in the control group (using basiliximab alone) stratified according to **a** all enrolled patients, **b** patients with grade 3–4 SR-aGVHD, **c** patients with skin SR-GVHD, **d** patients with gut SR-GVHD, **e** patients with liver SR-GVHD, and **f** patients with SR-GVHD involving ≥2 organs

The responses of SR-aGVHD in the two groups at week 4 are shown in Table 3. Patients in the MSC group received a median of 4 (range, 1–8) doses of MSC infusion. In the ITT population, the 4-week CR rate of SR-aGVHD in the MSC group proved to be significantly better than that in the control group (83.1% vs. 55.4%, *P* = 0.001). Regarding the aGVHD grade, patients with grade II–IV or grade III–IV SR-aGVHD displayed higher 4-week CR rates in the MSC group than in the control group (79.3% vs. 50.0%, *P<*0.001 for grade II–IV SR-aGVHD; 78.9% vs. 36.4%, *P*<0.001 for grade III–IV SR-aGVHD). Regarding the involved organs, the CR rates at week 4 in the MSC group were 83.6%, 78.6% and 50% for skin, gastrointestinal (GI) and liver SR-aGVHD, respectively, which were significantly higher than those in the control group. Among patients with SR-aGVHD involving ≥2 organs, the MSC group also achieved a favourable CR rate at week 4 (78.4% vs. 40.5%, *P* = 0.001). However, for the ORRs at week 4, the two groups showed comparable responses regardless of the degree of SR-aGVHD or the involved organs (Table 3 and Fig. 3).

**Table 3:** Treatment response of SR-aGVHD between the two groups at week 4

Outcomes	MSC group	Control group	*P*
CR rates of aGVHD grade, *n/n* (%)			
II-IV	54/65 (83.1%)	36/65 (55.4%)	**0.001**
III-IV	26/33 (78.9%)	11/33 (36.4%)	**< 0.001**
ORR of aGVHD grade, *n/n* (%)			
II-IV	61/65 (93.8%)	55/65 (84.6%)	0.09
III-IV	29/33 (87.9%)	25/33 (75.8%)	0.202
CR rates of aGVHD involved organs, *n/n* (%)			
Skin	46/55 (83.6%)	28/53 (52.8%)	**0.001**
GI	33/42 (78.6%)	22/48 (45.8%)	**0.001**
Liver	9/15 (60%)	3/17 (17.6%)	**0.014**
ORR of aGVHD according to involved organs, *n/n* (%)			
Skin	52/55 (94.5%)	45/53 (84.9%)	0.098
GI	39/42 (92.9%)	38/48 (79.2%)	0.065
Liver	11/15 (73.3%)	10/17 (58.8%)	0.388
CR rate of aGVHD involved ≥ 2 organs, *n/n* (%)	29/37 (78.4%)	15/37 (40.5%)	**0.001**
ORR of aGVHD involved ≥ 2 organs, *n/n* (%)	34/37 (91.9%)	28/37 (75.7%)	0.058

### MSCs may have helped to limit the organs involved in cGVHD

At week 52, cGVHD was observed in 35 patients in the MSC group and 39 patients in the control group. Among them, overlap syndrome occurred in 4 vs. 2 patients in the MSC and control groups, respectively (*P* = 0.34). Fewer patients in the MSC group developed cGVHD involving ≥3 organs (14.3% vs. 43.6%, *P* = 0.006). The 52-week cumulative incidences of all stages/moderate-severe/severe cGVHD were 52.3% (95% CI, 42.8–61.8%)/35.4% (95% CI, 27.3–43.6%)/14.1% (95% CI, 9.5–18.7%) in the MSC group and 58.5% (95% CI, 49.3–67.7%)/24.6% (95% CI, 17.6–31.7%)/9.2% (95% CI, 6.1–12.4%) in the control group, all of which were comparable between the two groups (*P* values were not shown).

### MSCs reduced the doses of basiliximab in the combination treatment

In the ITT population, patients received a median of 4 (range, 2 to 8) doses of basiliximab, and no significant difference was found in the number of basiliximab doses used between the two groups. However, more patients in the control group used ≥ 6 doses of basiliximab (4.6% (3/65) vs. 20% (13/65), *P* = 0.008).

### More patients could withdraw from CNIs

The median number of days of steroid treatment in the two groups was 16 (6–127) and 15 (5–202) after enrolment (*P* = 0.598), respectively. And most patients (61 in the MSC group vs. 61 in the control group) had steroid-free intervals. Finally, a total of 37 patients in the MSC group and 31 patients in the control group needed retreatment with steroids for aGVHD/cGVHD, poor engraftment function or cytokine release syndrome (CRS) (*P* = 0.291). The median steroid-free interval was 35 (2–357) days in the MSC group and 35 (3–362) days in the control group (*P* = 0.524).

During the 52-week follow-up, except for 10 patients in the MSC group and 8 patients in the control group who did not stop CNI treatment before death, significantly more patients in the MSC groups could withdraw from CNIs (81.8% (45/55) vs. 70.2% (33/57), *P* = 0.006). For patients with malignant haematological diseases, similar results were observed. Although the steroid-free interval was comparable between the two groups, more patients in the MSC group could discontinue CNIs (89.7% (35/39) vs. 63.6% (21/33), *P* = 0.004), except for 7 patients in the MSC group and 8 patients in the control group who did not stop CNI treatment before death.

### MSC-derived immunosuppressive cytokines increased after MSC infusion

**Fig. 4:**
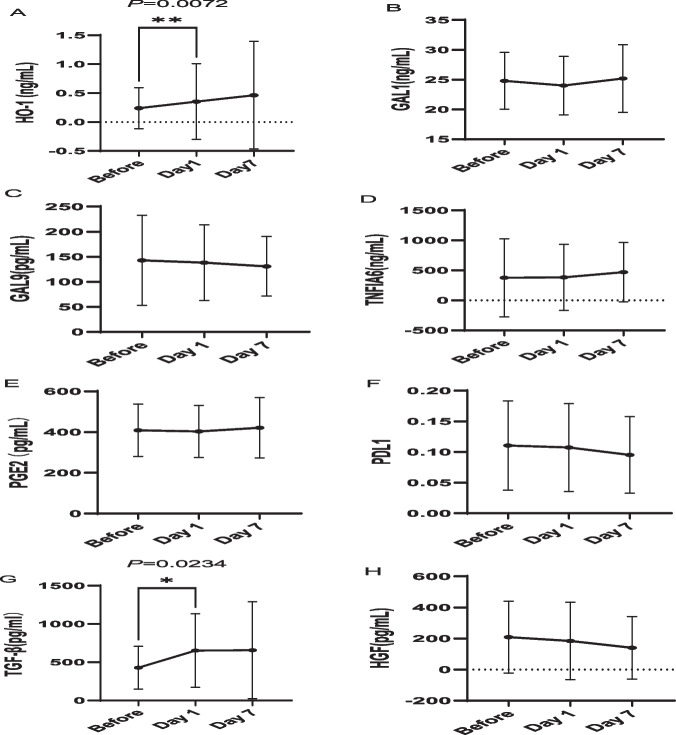
Changes in MSC-derived immunosuppressive cytokines after MSC infusion. **A**–**H** The levels of the MSC-derived immunosuppressive cytokines HO1, GAL1, GAL9, TNFIA6, PGE2, PDL1, TGF-β and HGF were assessed before and 1 day and 7 days after MSC infusion. Compared to the levels before MSC infusion, HO1 (**A**) and TGF-β (**G**) increased significantly 1 day after MSC infusion. At 7 days after MSC infusion, there was a trend towards increased levels of HO1 (**A**), GAL1 (**B**), TNFIA6 (**D**) and TGF-β (**G**), although the differences were not statistically significant

From March 1, 2022, to May 2, 2022, we collected blood samples from 19 consecutive patients enrolled in this trial from Peking University and evaluated MSC-derived immunosuppressive cytokines. The levels of HO1, GAL1, GAL9, TNFIA6, PGE2, PDL1, TGF-β and HGF are shown in Fig. 4. Compared to the levels before MSC infusion, HO1 (*P* = 0.0072) and TGF-β (*P* = 0.0243) levels increased significantly 1 day after MSC infusion. At 7 days after MSC infusion, there was a trend towards increased levels of HO1, GAL1, TNFIA6 and TGF-β, although the differences were not statistically significant.

### Toxicities and infections

In total, 62 patients in the MSC group and 65 in the control group were administered at least one dose of trial treatment, and they formed the safety population. MSCs were well tolerated, and there were no infusion-related AEs during or within 4 h after infusion. However, up to 28 days, most patients in both groups reported at least one AE. In both groups, the most frequent AE was infection, especially CMV infection, which occurred in 82.3% of the MSC group and 76.9% of the control group (*P* = 0.456) (Table 4). More patients in the control group suffered from EBV infection (13.8% vs. 3.2%, *P* = 0.033). All other AEs of infection, such as human herpesvirus-6 infection, bacterial infection and fungal infection, were comparable between the MSC group and control group. Specific AEs are shown in Additional file 3: Table S1.

**Table 4:** Most frequent adverse events up to week 4 (safety population)^*^

AEs	MSC group (*n* = 62)	Control group (*n* = 65)	*P*
Any AE	51 (82.3%)	50 (76.9%)	0.456
CMV infection	51 (82.3%)	50 (76.9%)	0.456
CMV disease	6 (9.7%)	6 (9.2%)	0.931
EBV infection	2 (3.2%)	9 (13.8%)	**0.033**
EBV disease	1 (1.6%)	5 (7.7%)	0.102
HHV-6 infection	5 (8.1%)	4 (6.2%)	0.675
Platelets decreased^a^	6 (9.7%)	5 (7.7%)	0.691
Neutropenia^a^	7 (11.3%)	11 (16.9%)	0.363
Anaemia^a^	5 (8.1%)	2 (3.1%)	0.218
Sepsis	4 (6.5%)	2 (3.1%)	0.370
Bacterial pneumonia	1 (1.6%)	4 (6.2%)	0.188
Fungal infection	1 (1.6%)	1 (1.5%)	0.973
Abdominal or intestinal infection	4 (6.5%)	9 (13.8%)	0.169
Acute kidney injury	4 (6.5%)	4 (6.2%)	0.945

*Abbreviations: AEs* adverse events, *CMV* cytomegalovirus, *EBV* Epstein–Barr virus, *HHV-6* human herpesvirus-6

^*^The safety population included all patients who received at least one dose of MSCs

^a^The new onset hematocytopenia after enrollment

### Survival

During the 52-week follow-up, death occurred in 11 patients in the MSC group at a median of 118 (13–300) days after enrolment and 14 patients in the control group at a median of 51 (18–323) days after enrolment (*P* = 0.317). All surviving patients completed the 52-week follow-up. The causes of death in the MSC and control groups included primary disease relapse (*n* = 1 vs. 2), aGVHD (*n* = 3 vs. 1), severe infections (*n* = 4 vs. 9), and transplant-associated thrombotic microangiopathy (*n* = 3 vs. 2). In our study, if patients had severe aGVHD refractory to treatments when they died, the cause of their death was defined as aGVHD. All 4 patients who died of aGVHD experienced infectious complications before death. Among them, 1 developed septic shock (*Enterococcus faecium*), 1 developed lung infection and 2 developed CMV enteritis. Three patients were refractory to experimental treatments at week 4. They stopped the experimental treatment and added salvage therapy for aGVHD; 2 added ruxolitinib plus etanercept and 1 added ruxolitinib plus faecal microbiota transplantation. However, they did not respond to the salvage therapy. The remaining patient achieved CR of aGVHD at week 4, but aGVHD relapsed after donor lymphocyte infusion (DLI) for the treatment of positive minimal residual disease at 6 months after enrolment. He died of aGVHD after DLI.

**Fig. 5:**
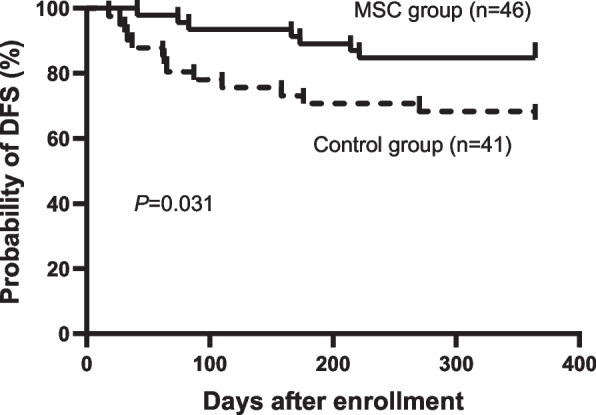
The 52-week disease-free survival (DFS) in patients with malignant haematological diseases after enrolment stratified according to the MSC group and control group

As the major secondary endpoints, OS at week 4, week 8, week 12, week 24 and week 52 was comparable between the two groups (Supplementary materials). The 52-week OS was 83.1% (95% CI 73.9–92.3%) in the MSC group and 78.5% (95% CI 68.5–88.5%) in the control group, which were comparable between the two groups (*P* = 0.460). However, patients with malignant haematological diseases (AML/ALL/CML) in the MSC group (*n* = 46) had a higher 52-week DFS than patients in the control group (*n* = 41) (84.8%, 95% CI 74.4–95.2% vs. 65.9%, 95% CI 51.4–80.4%, *P* = 0.031, Fig. 5). For patients with malignant haematological diseases, the incidences of relapse were similar (6.5% vs. 9.8%, *P* = 0.580), but more patients in the control group died of infection (4.3% vs. 17.1%, *P* = 0.052).

## Discussion

SR-aGVHD is associated with poor outcomes, and the 2-year mortality rates exceed 70% [1, 2]. However, an optimal therapy for SR aGVHD has not been established. This multicentre, randomized, phase 3 trial showed a significant improvement with basiliximab plus MSCs as the second-line therapy for SR-aGVHD after allo-HSCT. The findings of this trial clarified that basiliximab plus MSC therapy proved to have a significantly better 4-week CR rate than basiliximab alone and was well tolerated for SR-aGVHD.

According to EBMT GVHD management recommendations (2020) [1, 2], centres could choose second-line therapy following their institutional guidelines. Among the available second-line therapies for aGVHD, ruxolitinib is the only approved second-line therapy by the FDA, which led to 4-week OR rates of 55–62% and 4-week CR rates of 27–34% for SR-aGVHD in clinical trials. However, the toxic effect of a higher incidence of thrombocytopenia and virus infection might limit its application [7, 25]. ECP is a potentially effective therapy for SR-aGVHD via induction of immune effects directed against alloreactive T-cell populations without immunosuppression. Prospective studies have proven the effectiveness and safety of ECP for SR-aGVHD, especially for skin SR-aGVHD, with a response rate higher than 80% [36, 37]. However, ECPs are not available in China. The use of MMF for the treatment of refractory acute GVHD has been reported only in small-size studies [10, 38–41]. Furthermore, many novel monoclonal antibodies against T cells or cytokines involved in the development of aGVHD have attracted special attention. A randomized multicentre trial showed that a monoclonal antibody directed against CD3 (OKT3) plus high-dose methylprednisolone (HD-MP, 10 mg/kg) for SR-aGVHD had a higher ORR on study day 100 than HD-MP alone (53% vs. 33%; *P* = 0.06) [42]. Vedolizumab against α4β7 integrin [42], antibodies against TNF (infliximab) or the TNF receptor (etanercept) [43] are also potential therapies for SR-aGVHD. In addition, local intra-arterial steroids (IAS), which could give an organ-confined concentration and reduce the risk of long-term treatment side effects, may also be a second-line option for SR-aGVHD. A prospective large study of 120 patients assessed IAS treatment for steroid-resistant or steroid-dependent GVHD. The OR/CR of GI GVHD was 67.9% and 47.6%, whereas the hepatic OR/CR was 54.9% and 33.3%, respectively. However, its routine use may be limited by the complications associated with arterial catheterization, such as arterial rupture, dissection or thrombosis, temporary arterial spasm, groin haematoma or cellulitis and, rarely, renal failure [44].

In our centres, the standard second-line treatment for SR-aGVHD was basiliximab. In a prospective phase II trial, basiliximab for SR-aGVHD in 23 patients showed a primary overall response rate of 82.5% with a CR rate of 17.5% and a PR rate of 65% [9]. Other single-centre studies with a limited sample size have also identified the efficacy of basiliximab in SR-aGVHD [45–47]. Two relatively large, retrospective cohorts from our centre have reported that the ORR of basiliximab is approximately 80% in SR-aGVHD patients [33, 48]. A large-scale real-world analysis was performed, which involved a total of 940 patients with SR-aGVHD treated with basiliximab. The results showed that the cumulative incidence of ORR on day 28 was 79.4%, with a 3-year OS rate of 64.3% [49]. In our present study, the 4-week ORR and CR rate of basiliximab monotherapy were consistent with the outcomes of previous studies [33, 49]. The superior outcomes (ORR of 93.8% and CR rate of 83.1%) using MSCs plus basiliximab therapy had been achieved in the present trial, which might have benefited from the blockade of multiple effector pathways by the combination of basiliximab and MSCs in the treatment of SR-aGVHD.

Previous studies focusing on basiliximab or MSCs were retrospective and/or single-arm, and the small size may have reduced the ability to estimate the clinical effect of basiliximab or MSCs. Additionally, related haploidentical donors are an alternative option for patients without a fully matched sibling (MSD) or a well-matched unrelated donor (URD) for allo-HSCT. For HID HSCT, severe GVHD is a major barrier to successful HID HSCT, and the incidence is much higher than that in MSD or URD patients [29, 50]. SR-aGVHD in HID recipients warrants specific investigation. However, few randomized trials have included a sufficient number of HID HSCT recipients with SR-aGVHD. To our knowledge, this was the largest multicentre, prospective, randomized controlled clinical trial of MSCs for SR-aGVHD after HID HSCT. The results provide evidence for the clinical application of MSCs for SR-aGVHD, especially in HID HSCT.

Additionally, MSC therapy appears safe in this trial. MSC infusions were well tolerated without transfusion toxicity, and patients treated with MSCs plus basiliximab and basiliximab alone experienced similar rates of AEs. For the immune suppression related to MSCs, infections and relapse of the underlying disease were considered the most likely AEs related to treatment. In a previous study, MSCs and HSCs were transplanted in MSD HSCT to prevent GVHD. The results showed that cotransplantation of MSCs and HSCs obviously increased the relapse rate of underlying diseases [51]. However, an increase in infection or relapse has not been observed in other studies on MSCs in the treatment of GVHD [22, 52–54]. The current trial similarly found no trend for increased infection or relapse rates in the MSC group, and the infection rate was even reduced in the MSC group with malignant diseases. The shorter duration of steroid treatment in this trial (a median of 15 or 16 days after enrolment for the two groups, respectively) may have led to the lower rate of severe infection compared to that in the REACH2 study [22, 52–54], in which only 21% of patients in the ruxolitinib group and 14% of patients in the control group had discontinued steroids by day 56. However, previous studies have found that MSCs possess direct antimicrobial activity [55], which might explain the decreased infection in the MSC group. Significantly more patients withdrew from CNIs within 52 weeks, and less basiliximab was used in the MSC group. The reduced immunosuppression may also contribute to the reduced infection and improved 52-week DFS in the MSC group with malignant haematological disease. However, further proof is needed.

The cumulative incidence of cGVHD in a series of studies of HID HSCT for haematological malignancies in our centre ranged from 42.3 to 63.3% [56, 57], which was higher than that in MSD HSCT [29]. SR-aGVHDs are regarded as significant independent risk factors for the development of cGVHD after allo-HSCT [58]. All patients in our study had SR-aGVHD, and most received HID HSCT, which may suggest a higher incidence of cGVHD in our present study. Although the incidences of cGVHD were comparable between the two groups, fewer patients in the MSC group developed cGVHD involving ≥3 organs in our present study, which may be attributed to the higher early CR rate after MSC infusions. However, this remains an important area of investigation.

Since MSCs cannot secrete granzymes or perforins and produce antibodies, these cells lack direct cytotoxic or humoral defence activity. After the transfusion, MSCs do not persist, and the majority die within 48 h [59–62]. They work by producing extracellular vesicles (EVs) and secreting cytokines, chemokines and growth factors that can signal to other cells and tissues, ultimately leading to immunosuppression [63–66]. However, few reports have shed light on the changes in the cytokine spectrum before and after MSC transfusion during the treatment of SR aGVHD. In this study, the MSC-derived suppressive cytokines HO1, GAL1, GAL9, TNFIA6, PGE2, PDL1, TGF-β and HGF were measured. Our findings first showed that HO-1 and TGF-β were significantly increased 1 day after MSC infusion, which might offer insight into the potential mechanism of MSC therapy for SR-aGVHD. However, the other cytokines derived from MSCs were not significantly different before and after MSC infusion, which might have been related to the sample size in our study. Further studies with larger sample sizes are warranted to support this conclusion.

The major limitation of the present study is the relatively short follow-up time, which may have led to a higher risk of bias in the evaluation of the incidences of infection, cGVHD, relapse and survival. Further results of long-term follow-up should be reported. Another limitation of this study is that we measured soluble factors in only 19 patients in the MSC group, as most patients were not willing to offer blood samples for testing. It is also regrettable that we did not measure cytokine levels in patients in the control arm and could not compare changes in suppressive mediators between the two groups. Since IL-2 receptor blockade could reverse the inflammatory environment in aGVHD, the suppressive mediators might increase just by modulating acute GVHD with IL2-R blockade. However, a significant depletion of the regulatory T-cell subset was found in patients with SR-aGVHD following basiliximab treatment, which might be because basiliximab-mediated IL-2R blockade removes the stimulatory factor needed for regulatory T cells [47]. As regulatory T cells can release various suppressive mediators, such as TGF-β and IL-10, it is difficult to evaluate the regulatory cytokine profile induced by IL2-R blockade alone. Further study is needed to explore the change in suppressive mediators under MSC and IL-2R blockade. A comparative study between groups on the cytokine spectrum after MSCs ± basiliximab infusion appears to be warranted. Finally, our trial was limited by the majority of haplo-HSCT patients using an ATG-based regimen as anti-GVHD prophylaxis, and the conclusions of this trial might not be applicable to PTCy-based haplo-HSCT, which has been widely used in the US/Europe. A further trial is needed to study the efficacy and safety of MSCs plus basiliximab in PTCy-based haplo-HSCT.

## Conclusions

This trial shows that for patients with SR-aGVHD after allo-HSCT, especially HID HSCT, the combination of MSCs with basiliximab as the second-line therapy led to significantly better 4-week CR rates than treatment with basiliximab alone. The addition of MSCs not only did not increase toxicity, such as infection and relapse, but also brought a survival benefit. This trial provides compelling evidence for the efficacy and safety of MSC therapy for SR-aGVHD to establish the role of MSC therapy in the treatment of this condition.

### Supplementary Information

**Additional file 1.** Protocol for the prevention of infections.

**Additional file 2.** Protocol for the prevention of graft-versus-host disease (GVHD).

**Additional file 3:** **Table S1.** CTCAE Grade of adverse events(AEs).

## Data Availability

The authors declared that all and the other data supporting the findings of this study are available within the paper. The raw data that support the findings of this study are available from the corresponding author upon reasonable request.

## Abbreviations

aGVHD: Acute graft-versus-host disease
AEs: Adverse events
allo-HSCT: Allogeneic haemopoietic stem cell transplantation
cGVHD: Chronic GVHD
CIs: Confidence intervals
CsA: Cyclosporine A
DFS: Disease-free survival
DLI: Donor lymphocyte infusion
ELISA: Enzyme-linked immunosorbent assay
Gal-1: Galectin-1
Gal-9: Galectin-9
GI: Gastrointestinal
HID HSCT: Haploidentical haemopoietic stem cell transplantation
HR: Hazard ratios
HO-1: Haem oxygenase-1
HGF: Hepatocyte growth factor
ITT: Intention-to-treat
MSD: Matched sibling
MSCs: Mesenchymal stromal cells
mITT: Modified ITT
NR: No response
OR: Overall response
OS: Overall survival
PR: Partial response
PTCy: Posttransplant cyclophosphamide
PDL1: Programmed death ligand 1
PGE2: Prostaglandin E2
SR-aGVHD: Steroid-refractory acute graft-versus-host disease
TSG-6: TNFα stimulated gene 6
TGF-β1: Transforming growth factor
UC-MSCs: Umbilical cord blood
URD: Unrelated donor
