# Supplementary material for: Mesenchymal stromal cells plus basiliximab improve the response of steroid-refractory acute graft-versus-host disease as a second-line therapy: a multicentre, randomized, controlled trial
Source: BMC Med. 2024 Feb 27;22:85. doi: 10.1186/s12916-024-03275-5 (PMC10900595; doi:10.1186/s12916-024-03275-5)
Supplement: Supplementary file 1 — Additional file 1. Protocol for the prevention of infections. [file 12916_2024_3275_MOESM1_ESM.docx]

**Supplementary materials**

**Additional file 1: Protocol for the prevention of infections**

All patients were hospitalized in rooms with high-efficiency particlearresting air filters during conditioning.Patients received oral norfloxacin from the start of conditioning until neutrophil engraftment and intravenous piperacillin or cephalosporin during the neutropenia period for bacteria prophylaxis.As prophylaxis for pneumocystis, trimethoprim (TMP)/sulfamethoxazole (SMZ) was administered orally every day at a dose of 5 mg/kg/d TMP from day -10 to day +30 and then was administered orally 2 days per week at a dose of 5 mg/kg/d TMP from day +30 to day +180. Ganciclovir was given for cytomegalovirus prophylaxis from the start of conditioning to day -2, and acyclovir was given for herpes simplex virus prophylaxis from day +1 to day +365. Prophylaxis for fungal infection included primary prophylaxis for patients without a prior history of invasive fungal infection (IFD) and secondary prophylaxis for patients with a prior history of IFD. Posaconazole was administered from the start of conditioning to day +75 as primary prophylaxis,for patients with aplastic anemia caspofungin replaced posaconazole as primary prophylaxis during conditioning.Secondary prophylaxis including the effective antifungals previously was administrated from the start of conditioning to day +100.
