# Supplementary material for: Mesenchymal stromal cells plus basiliximab improve the response of steroid-refractory acute graft-versus-host disease as a second-line therapy: a multicentre, randomized, controlled trial
Source: BMC Med. 2024 Feb 27;22:85. doi: 10.1186/s12916-024-03275-5 (PMC10900595; doi:10.1186/s12916-024-03275-5)
Supplement: Supplementary file 2 — Additional file 2. Protocol for the prevention of graft-versus-host disease (GVHD). [file 12916_2024_3275_MOESM2_ESM.docx]

**Supplementary materials**

**Additional file 2: Protocol for the prevention of graft-versus-host disease (GVHD)**

Cyclosporine A (CsA) plus short-term methotrexate(MTX)and mycophenolate mofetil (MMF) were administered for the prevention of GVHD. On Day +1, MTX (15 mg/m^2^) was administered intravenously and then 10 mg/m^2^ were given on Days +3, +6 and +11 after transplantation. Four doses of MTX were given to HID and URD transplantation patients while only the first three doses were given to MSD transplantation patients. Intravenous cyclosporine (2.5 mg/kg twice a day) was started on day −9 and continued until patients were able to tolerate oral medication. Thereafter, CsA was given orally twice daily with target trough levels of 150–250 ng/mL. MMF (500mg twice daily) was begun on day −9 and discontinued on the day of neutrophil engraftment.
