# Supplementary material for: Mesenchymal stromal cells plus basiliximab improve the response of steroid-refractory acute graft-versus-host disease as a second-line therapy: a multicentre, randomized, controlled trial
Source: BMC Med. 2024 Feb 27;22:85. doi: 10.1186/s12916-024-03275-5 (PMC10900595; doi:10.1186/s12916-024-03275-5)
Supplement: Supplementary file 3 — Additional file 3: Table S1. CTCAE Grade of adverse events(AEs). [file 12916_2024_3275_MOESM3_ESM.docx]

**Supplementary materials**

**Additional file 3: Table S1. CTCAE Grade of adverse events(AEs)**

| AEs | MSC group (n=62) | Control group (m=65) | P |
| --- | --- | --- | --- |
| Any AEs | 51(82.3%) | 50(76.9%) | 0.456 |
| CMV infection |  |  |  |
| Grade 1 | 0(0%) | 0(0%) | - |
| Grade 2 | 45(72.6%) | 44(67.7%) | 0.548 |
| Grade 3 | 6(9.7%) | 6(9.2%) | 0.931 |
| Grade 4 | 0(0%) | 0(0%) | - |
| EBV infection |  |  |  |
| Grade 1 | 0(0%) | 0(0%) | - |
| Grade 2 | 2(3.2%) | 9(13.8%) | 0.033 |
| Grade 3 | 1(1.6%) | 5(7.7%) | 0.102 |
| Grade 4 | 0(0%) | 0(0%) | - |
| HHV-6 infection |  |  |  |
| Grade 1 | 0(0%) | 0(0%) | - |
| Grade 2 | 5(8.1%) | 4(6.2%) | 0.675 |
| Grade 3 | 0(0%) | 0(0%) | - |
| Grade 4 | 0(0%) | 0(0%) | - |
| Platelets decreased^a^ |  |  |  |
| Grade 1 | 1(1.6%) | 1(1.5%) | 0.973 |
| Grade 2 | 2(3.2%) | 2(3.1%) | 0.962 |
| Grade 3 | 2(3.2%) | 1(1.5%) | 0.531 |
| Grade 4 | 1(1.6%) | 1(1.5%) | 0.973 |
| Neutropenia^a^ |  |  |  |
| Grade 1 | 2(3.2%) | 4(6.2%) | 0.437 |
| Grade 2 | 2(3.2%) | 3(4.6%) | 0.687 |
| Grade 3 | 2(3.2%) | 2(3.1%) | 0.962 |
| Grade 4 | 1(1.6%) | 2(3.1%) | 0.587 |
| Anemia^a^ |  |  |  |
| Grade 1 | 1(1.6%) | 0(0%) | 0.304 |
| Grade 2 | 1(1.6%) | 0(0%) | 0.304 |
| Grade 3 | 3(4.8%) | 1(1.5%) | 0.287 |
| Grade 4 | 1(1.6%) | 1(1.5%) | 0.973 |
| Sepsis |  |  |  |
| Grade 1 | 0(0%) | 0(0%) | - |
| Grade 2 | 0(0%) | 0(0%) | - |
| Grade 3 | 4(6.4%) | 0(0%) | 0.037 |
| Grade 4 | 0(0%) | 2(3.1%) | 0.164 |
| Bacterial pneumonia |  |  |  |
| Grade 1 |  |  |  |
| Grade 2 |  |  |  |
| Grade 3 | 1(1.6%) | 4(6.2%) | 0.188 |
| Grade 4 | 0(0%) | 0(0%) | - |
| Fungal infection |  |  |  |
| Grade 1 | 0(0%) | 0(0%) | - |
| Grade 2 | 0(0%) | 0(0%) | - |
| Grade 3 | 1(1.6%) | 0(0%) | 0.304 |
| Grade 4 | 0(0%) | 1(1.5%) | 0.327 |
| Abdominal or intestinal infection |  |  |  |
| Grade 1 | 0(0%) | 0(0%) | - |
| Grade 2 | 0(0%) | 0(0%) | - |
| Grade 3 | 4(6.4%) | 8(12.3%) | 0.259 |
| Grade 4 | 0(0%) | 1(1.5%) | 0.327 |
| Acute kidney injury |  |  |  |
| Grade 1 | 3(4.8%) | 4(6.2%) | 0.745 |
| Grade 2 | 1(1.6%) | 0(0%) | 0.304 |
| Grade 3 | 0(0%) | 0(0%) | - |
| Grade 4 | 0(0%) | 0(0%) | - |
